# Fetal heart rate variability is a biomarker of rapid but not progressive exacerbation of inflammation in preterm fetal sheep

**DOI:** 10.1038/s41598-022-05799-3

**Published:** 2022-02-02

**Authors:** Shoichi Magawa, Christopher A. Lear, Michael J. Beacom, Victoria J. King, Michi Kasai, Robert Galinsky, Tomoaki Ikeda, Alistair J. Gunn, Laura Bennet

**Affiliations:** 1 Fetal Physiology and Neuroscience Group, Department of Physiology, The University of Auckland, Auckland, New Zealand; 2 Department of Obstetrics and Gynecology, Mie University, Mie, Japan; 3 Department of Obstetrics and Gynecology, Yokohama City University, Yokohama, Japan; 4 The Ritchie Centre, Hudson Institute of Medical Research and Department of Obstetrics and Gynaecology, Monash University, Clayton, Australia; 5 Department of Physiology, Faculty of Medical and Health Sciences, The University of Auckland, Private Bag 92019, Auckland, 1142 New Zealand

**Keywords:** Biomarkers, Predictive markers, Experimental models of disease, Preclinical research

## Abstract

Perinatal infection/inflammation can trigger preterm birth and contribute to neurodevelopmental disability. There are currently no sensitive, specific methods to identify perinatal infection. We investigated the utility of time, frequency and non-linear measures of fetal heart rate (FHR) variability (FHRV) to identify either progressive or more rapid inflammation. Chronically instrumented preterm fetal sheep were randomly assigned to one of three different 5d continuous i.v. infusions: 1) control (saline infusions; n = 10), 2) progressive lipopolysaccharide (LPS; 200 ng/kg over 24 h, doubled every 24 h for 5d, n = 8), or 3) acute-on-chronic LPS (100 ng/kg over 24 h then 250 ng/kg/24 h for 4d plus 1 μg boluses at 48, 72, and 96 h, n = 9). Both LPS protocols triggered transient increases in multiple measures of FHRV at the onset of infusions. No FHRV or physiological changes occurred from 12 h after starting progressive LPS infusions. LPS boluses during the acute-on-chronic protocol triggered transient hypotension, tachycardia and an initial increase in multiple time and frequency domain measures of FHRV, with an asymmetric FHR pattern of predominant decelerations. Following resolution of hypotension after the second and third LPS boluses, all frequencies of FHRV became suppressed. These data suggest that FHRV may be a useful biomarker of rapid but not progressive preterm infection/inflammation.

## Introduction

Preterm birth accounts for about 11% of deliveries worldwide^1^ and is a leading cause of neonatal morbidity and mortality^2^. Perinatal infection and inflammation are associated with approximately 40% of premature deliveries^3–7^, and high rates of neurodevelopmental disability^8,9^. In recent studies, gram-negative bacteria were predominantly involved in early-onset sepsis amongst preterm neonates, denoting a high rate of gram-negative infection in the immediate perinatal period^10^. Further, the contribution from gram-negative *Escherichia coli* infections in very low-birth weight infants has increased markedly in recent years^10^. Diagnosis of perinatal infection still largely relies on maternal symptoms, but milder or more slowly evolving infections are typically asymptomatic^11^. There is evidence that acute onset of intrauterine infection may be associated with fetal heart rate (FHR) changes, including tachycardia and loss of accelerations and fetal heart rate variability (FHRV)^12^, but there is limited systematic information.

Sensitive markers of fetal exposure to infection are urgently needed to achieve early medical intervention for fetuses exposed to infection while also avoiding unnecessary interventions. In preterm neonates, changes in heart rate variability (HRV) can allow early detection of sepsis before clinical deterioration, facilitating earlier treatment and improved survival^13–16^. The key heart rate features of neonatal infection include asymmetrical heart rate traces, reduced time domain measures of HRV, with reduced sample entropy and measures of nonlinear HRV^13,15,17^. The combined analysis of these measures into a single score called the heart rate characteristic index has recently been reported to have an overall sensitivity of 53% and specificity of 80% for culture-proven late-onset neonatal sepsis across all gestational ages^18^. Moreover, a higher sensitivity of 76% but specificity of 63% was found among infants born < 28 weeks^18^, supporting a moderate ability to identify sepsis among extremely preterm infants.

The inflammatory response to infection, rather than the infection per se, is the crucial damaging event to the developing brain^19^. Thus, lipopolysaccharide (LPS), a component of the cell wall of gram-negative bacteria, is widely used to induce systemic and central inflammation^20^. For example, fetal sheep exposed to acute severe inflammation induced by high-dose lipopolysaccharide (LPS), a component of the cell wall of gram-negative bacteria, develop transient hypotension with biphasic changes in time domain measures of FHRV characterized by an initial increase followed by suppression of FHRV^21,22^. By definition, these studies involved very rapid increases in the inflammatory stimulus after boluses of LPS. The relationship between the speed of onset of fetal infection/inflammation and changes in FHRV remains highly unclear.

In the present study, we contrasted changes in FHRV during two different patterns of fetal inflammation. Firstly, we utilized a pattern of acute on chronic LPS infusions to represent a slowly evolving fetal infection with acute severe exacerbations^23^. We compared this to a second pattern of progressively increased LPS infusions designed to represent progressively worsening fetal inflammation without acute exacerbations^24^. We investigated changes in a wide range of FHRV measures, including time and frequency domain analysis, as well as non-linear measures, including measures that have been reported to predict acute neonatal sepsis^13^.

## Materials and methods

### Ethical approval

All procedures were approved by the Animal Ethics Committee of the University of Auckland following the New Zealand Animal Welfare Act 1999, and the Code of Ethical Conduct for animals in research established by the Ministry of Primary Industries, Government of New Zealand. All methods were performed in accordance with the relevant guidelines and regulations. This manuscript complies with the ARRIVE guidelines^25^.

### Surgical procedures

25 Romney/Suffolk fetal sheep were operated on at 97–101 days gestational age (term = 147 days)^23,24^. Food, but not water was withdrawn 18 h before surgery. Ewes were given long acting oxytetracycline (20 mg/kg, Phoenix Pharm Distributors, Auckland, New Zealand) intramuscularly 30 min before the start of surgery. Anesthesia was induced by intravenous injection of propofol (5 mg/kg; AstraZeneca, Auckland, New Zealand), and general anesthesia maintained using 2–3% isoflurane (Medsource, Ashburton, New Zealand) in oxygen. All surgical procedures were performed using sterile techniques. Polyvinyl catheters were placed in the left femoral artery and amniotic sac to measure MAP and amniotic pressure, respectively. Further catheters were placed in the right brachial artery and left femoral vein to allow for pre-ductal arterial blood sampling and intravenous infusions of LPS respectively. A pair of electrodes was sewn over the fetal chest to record the fetal electrocardiogram (ECG). Another pair of electrodes was sewn into nuchal muscle to measure nuchal electromyographic (EMG) activity. All fetal leads were exteriorized through the maternal flank and a maternal long saphenous vein was catheterized to provide access for postoperative care and euthanasia. 80 mg gentamicin (Pfizer Ltd, Auckland, New Zealand) was administered into the amniotic sac before closure of the uterus.

### Post-operative care

All sheep were housed in separate metabolic cages with access to water and food ad libitum, together in a temperature-controlled room (16 ± 1 °C, humidity 50 ± 10%) with a 12-h light/dark cycle. 4–5 days post-operative recovery was allowed before experiments, during which time antibiotics were intravenously administered to the ewe daily (600 mg benzylpenicillin sodium; Novartis, Auckland, New Zealand, and 80 mg gentamicin, Pfizer). Fetal catheters were maintained by continuous infusion of heparinized saline (20 U/ml at 0.2 ml/h).

### Experimental recordings

Fetal mean arterial blood pressure (MAP) corrected for maternal movement by subtraction of amniotic fluid pressure (Novatrans II, MX860; Medex Inc., Hilliard, OH, USA), ECG and EMG were recorded continuously throughout the experimental period. The blood pressure signal was collected at 64 Hz and low pass filtered at 30 Hz. The raw ECG signal was analogue filtered with a first-order high-pass filter set at 1 Hz and an eight-order low-pass Bessel filter set at 100 Hz and saved at 1024 Hz and used to derive FHR and FHRV, as described below. The nuchal EMG signal was band-pass filtered between 100 Hz and 1 kHz and the signal was integrated using a time constant of 1 s.

### Experimental protocol

**Figure 1:**
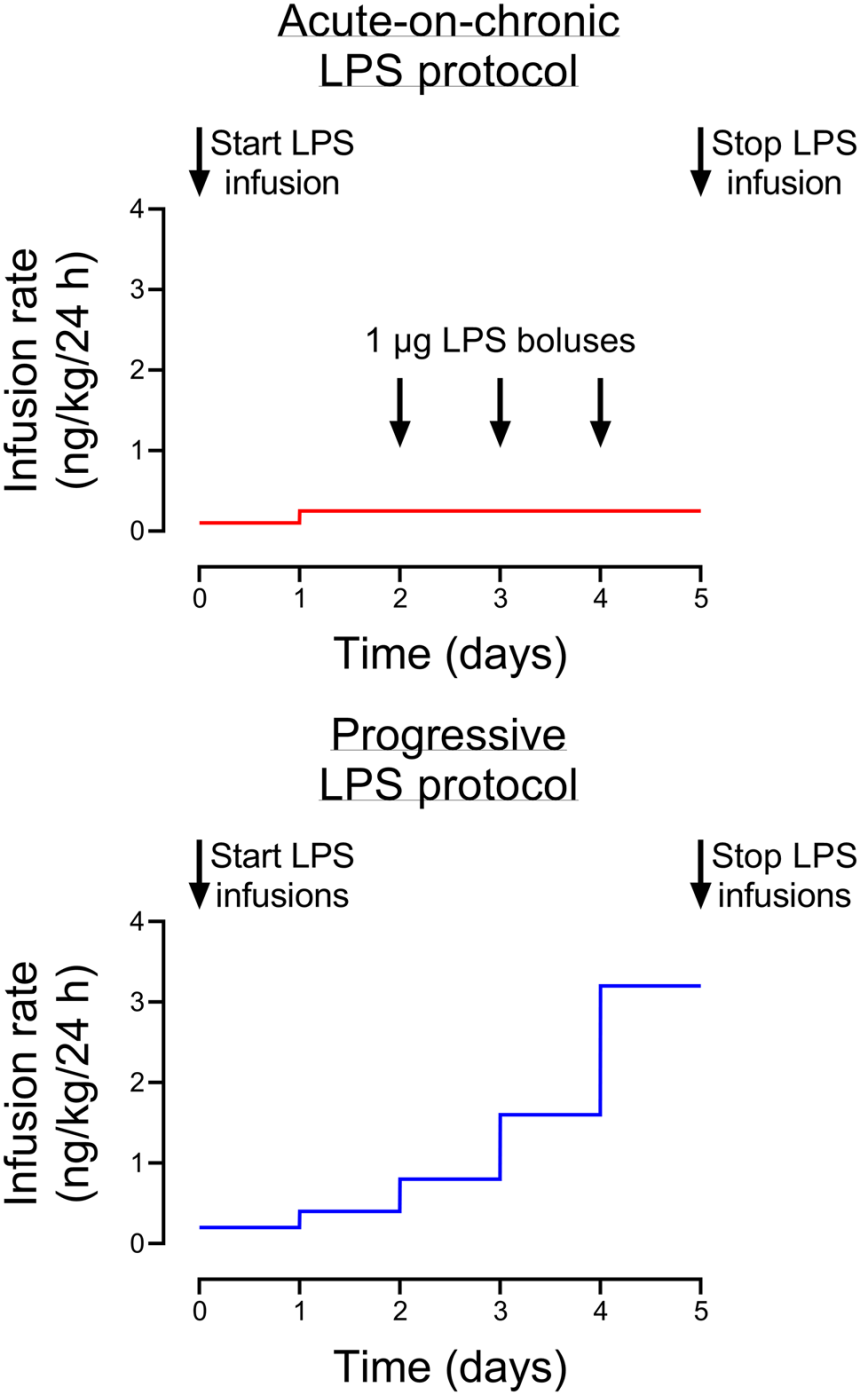
Schematic of the two LPS protocols. All LPS infusions were started on day 0 and continued for a total of 5 days, fetuses were studied for a further 5 days after the end of infusions. The progressive LPS group received LPS infusions which were doubled in dosage rate every 24 h. The acute-on-chronic LPS group received a low-dose LPS infusion, which was increased after 24 h and remained constant for the following 4 days. The acute-on-chronic LPS group additionally received three high-dose 1 µg LPS boluses, infused over 2 min, at 48, 72 and 96 h after the start of low-dose infusions.

Experiments were performed at 104–105 d (0.7) gestation. The preterm fetal sheep at 0.7 gestation is neurologically comparable to the human brain at 28–32 weeks of gestation^26^, before the onset of cortical myelination^27^. These studies used LPS derived from Escherichia coli, serotype 055:B5 (Sigma Aldrich, St. Louis, MO, USA). Fetuses were randomly assigned to one of three infusion protocols, each lasting a total of 5 days (120 h). The LPS protocols are shown in Fig. 1. LPS doses were calculated based on an estimated 1 kg fetal body weight at the start of the experiment.

1. Control (n = 10). Intravenous saline infusions and boluses at the same rate and volume as group 2.
2. Progressive LPS (n = 8). LPS was dissolved in saline at a concentration of 200 ng/mL; note that a higher concentration than used in group 3 was required in order to reduce the total volume to be infused. LPS was initially infused at a rate of 0.2 µg/kg/24 h (41.7 µL/h). The rate of LPS infusion was doubled every 24 h: 0.4 µg/kg/24 h (83.3 µL/h), 0.8 µg/kg/24 h (166.7 µL/h), 1.6 µg/kg/24 h (333.3 µL/h) and 3.2 µg/kg/24 h (666.7 µL/h)^24^. The total LPS dose given at the end of the experiment was 6.2 µg/kg in each fetus.
3. Acute on chronic LPS (n = 9). LPS was dissolved in saline at a concentration of 50 ng/mL, and initially infused at a rate of 0.1 µg/kg/24 h (83.3 µL/h) from 0–24 h of the experiment. The rate was then increased to 0.25 µg/kg/24 h (207.5 µL/h) from 24 to 120 h of the experiment. Additional bolus infusions of 1 µg LPS dissolved in 2 ml of saline (infused over 2 min) were performed at 48, 72 and 96 h after the start of LPS infusions^23^. On each day that a bolus was given, each fetus received a total of 1.25 µg/kg of LPS. The total LPS dose given at the end of the experiment was 4.1 µg/kg in each fetus.

All infusions were stopped after 120 h, and fetuses were studied for a further 120 h after the end of infusions. 240 h after the start of infusions, sheep were killed by intravenous injection of an overdose (9 g) of pentobarbital sodium (Pentobarb 300, Chemstock international, Christchurch, New Zealand).

### Fetal arterial blood samples

Fetal arterial blood was collected every morning starting from 24 h before the experiment until the day of postmortem. Baseline samples were taken 30 min before the rate of infusion of LPS was increased or a bolus was given. On the days of LPS infusions (day 0–4), additional samples were taken at 2 and 6 h after infusion rates were increased or boluses were administered. Whole blood samples were analyzed for fetal pH and blood gases (ABL800 blood gas analyzer, Radiometer, Copenhagen, Denmark) and for glucose and lactate measurements (YSI model 2300, Yellow Springs, OH, USA). Plasma samples at these time-points were collected for cytokine and endocrine analysis, as previously reported^21,23,24,28,29^.

### Data analysis

Off-line analysis of the physiological data was performed using customized LabVIEW-based programs (National Instruments). All measures were averaged in 1-h epochs during experimental period. Nuchal EMG data was normalized by using the mean value of the baseline period.

FHRV analysis was performed using the PhysioNet Cardiovascular Signal Toolbox^30^ for Matlab (MATLAB 2020A, The MathWorks, USA). All metrics were assessed on continuous, non-overlapping 5-min windows unless otherwise specified^31^. Time domain measures of FHRV were assessed as the standard deviation of RR intervals (SDNN) and the root-mean-square of successive differences in R-R interval (RMSSD). Frequency domain measures were obtained by calculation of the Lomb-Scargle periodogram. The frequency band boundaries used in this study were: very-low-frequency (VLF): 0.0033–0.04 Hz, low-frequency (LF): 0.04–0.15 Hz, high-frequency (HF): 0.15–0.4 Hz, very-high-frequency (VHF): 0.4–1.5 Hz^32–34^. The absolute spectral power of each band was natural log-transformed. In this study, we chose to utilize the standard adult VLF, LF and HF bands, and included an additional VHF band to interrogate higher frequency activity in view of evidence that FHRV includes higher frequency rhythms than the adult heart rate, at least in part due to the high frequency of fetal breathing movements^35–37^. Our rationale for the inclusion of an additional VHF band instead of adopting the proposed extended ‘fetal’ HF band is that this approach provides greater granularity of information, and allows better comparison to adult data^38,39^.

The non-linear index, Sample Entropy was calculated with parameters: embedding dimension m = 2 and tolerance r = 0.15 and maximum scale τ = 20. Acceleration capacity and deceleration capacity were calculated from the phase-rectified signal averaging algorithm^40^ using the following parameters: *L* = 45, *T* = 5, *s* = 2, based on previous studies^41^. As we have recently described^42^, simplistically every RR interval that increased (or decreased) relative to the previous RR interval was defined as a “acceleration anchor” (or “deceleration anchor”). A window of 2*L* (i.e. 90 RR intervals) was centred around every anchor, consisting of the anchor itself ‘x(0)’, the 45 data-points immediately following the anchor ‘x(1), x(2), … x(45)’ and the 44 data-points immediately preceding the anchor ‘x(−44), x(−43), … x(−1), x(0)’. All windows within the analysis epoch are then aligned at the anchor (windows are thereby ‘phase-rectified’). Each set of data-points within the aligned windows are then averaged to calculate $\overline{x}$(−44), $\overline{x}$(−43), …$\overline{x}$(45). Acceleration capacity and deceleration capacity are calculated separately as:

$$\frac{{[\bar{x}\left( { - 44} \right) + \bar{x}\left( { - 43} \right) + \cdots \bar{x}\left( 0 \right)] - [\bar{x}\left( 1 \right) + \bar{x}\left( 2 \right) + \cdots \bar{x}\left( {45} \right)]}}{{2L}}$$

Sample asymmetry was calculated as described by Kovatchev and colleagues^43^. Briefly, the median RR interval for each epoch is determined and then the raw RR intervals are separated into those greater and those less than the median. For RR intervals less than the median, the deviation of each RR interval from the median is calculated and then squared before the average of these values over the epoch is found. This final average is called R1. The same process is repeated for all RR intervals greater than the median, and the final average is called R2. Sample asymmetry is the ratio of R2/R1^43^. Sample Asymmetry was calculated from epochs consisting of 4096 consecutive RR intervals (approximately 20 min of data)^44^. Each epoch was selected from the start of every 30 min of recording and then converted into an hourly average, as previously described^22^.

### Statistical analysis

Statistical analysis was performed using SPSS (v25, IBM, Armonk, NY). The data were treated as two separate studies. First, we compared the control group with the acute-on-chronic LPS group and secondly we compared the control group with the progressive LPS group. The effect of each infusion protocol was separately compared by two-way ANOVA with time treated as a repeated measure. Statistical significance was accepted at P < 0.05. Data are presented as mean ± SEM.

## Results

### Previously published results

Results from overlapping cohorts included in this study have been previously published, including neurohistological^23,24,28,29^, magnetic resonance imaging-based^24^, cardiovascular^21–24,29^, neurophysiological^21,23,24^, and cytokine and endocrine outcomes^21,23,24,28,29^. Time-domain based FHRV analysis from the acute-on-chronic group have previously been published^21,22^.

### Baseline parameters and fetal biochemistry

**Figure 2:**
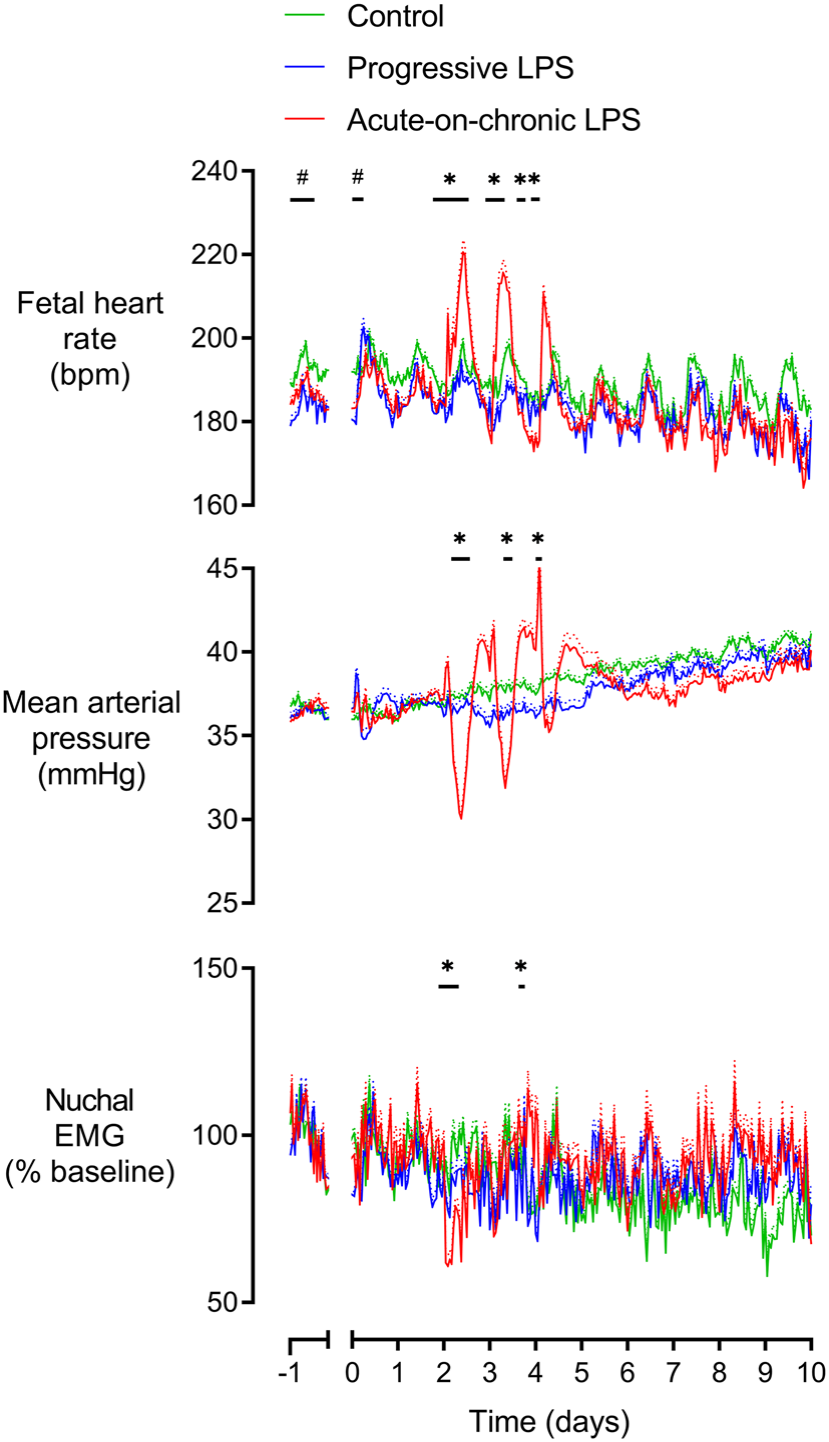
Changes in mean arterial pressure, fetal heart rate and nuchal electromyographic (EMG) activity during and after LPS infusions. Nuchal EMG activity is shown relative to baseline. All LPS infusions were started on day 0 and continued for a total of 5 days, fetuses were studied for a further 5 days after the end of infusions. Groups shown are control (green, n = 10), progressive LPS (blue, n = 8) and acute-on-chronic LPS (red, n = 9). Data are shown as 1 h mean ± SEM (mean is displayed as solid lines, SEM is displayed as dotted lines). Baseline FHR was included as a covariate. *P < 0.05 control vs. acute-on-chronic LPS, ^#^P < 0.05 control versus progressive LPS.

All fetuses in the present study were healthy based on our laboratory standards prior to the start of infusions, including normal arterial biochemistry and physiological parameters. There were no physiological, FHR or biochemical differences in the baseline period between the control and LPS groups, except that the progressive LPS group had a slightly higher baseline FHR compared with the control group (p = 0.043, Fig. 2). Baseline FHR was therefore included as a covariate in the statistical analysis. There were only minor differences between groups in fetal biochemistry during or after LPS infusions (Tables 1 and 2). There were no differences in fetal sex between the groups: control (4 females, 6 males), progressive LP (4 females, 4 males, acute on chronic LPS (5 females, 4 males).

**Table 1:** Arterial biochemistry from day 0 to day 3.

Day	Day 0			Day 1			Day 2			Day 3		
Time	Baseline	+ 2 h	+ 6 h	Baseline	+ 2 h	+ 6 h	Baseline	+ 2 h	+ 6 h	Baseline	+ 2 h	+ 6 h
**pH**												
Control	7.36 ± 0.00	7.36 ± 0.00	7.36 ± 0.00	7.35 ± 0.00	7.35 ± 0.01	7.35 ± 0.01	7.34 ± 0.00	7.34 ± 0.00	7.34 ± 0.01	7.34 ± 0.00	7.35 ± 0.01	7.35 ± 0.01
Acute-on-chronic	7.36 ± 0.00	7.36 ± 0.00	7.34 ± 0.01	7.35 ± 0.00	7.34 ± 0.00	7.35 ± 0.00	7.34 ± 0.00	7.30 ± 0.00*	7.32 ± 0.00	7.34 ± 0.00	7.29 ± 0.01*	7.34 ± 0.00
Progressive	7.36 ± 0.00	7.36 ± 0.00	7.36 ± 0.00	7.35 ± 0.00	7.35 ± 0.00	7.35 ± 0.00	7.35 ± 0.00	7.36 ± 0.00	7.34 ± 0.00	7.33 ± 0.00	7.35 ± 0.00	7.34 ± 0.00
**pCO**_**2**_** (mmHg)**												
Control	46.6 ± 0.3	46.2 ± 0.3	46.0 ± 0.3	47.5 ± 0.3	47.4 ± 1.2	47.8 ± 1.3	47.6 ± 0.3	47.2 ± 0.5	43.1 ± 2.9	46.4 ± 0.2	48.6 ± 0.7	48.7 ± 0.7
Acute-on-chronic	48.1 ± 0.5	48.4 ± 0.7	46.8 ± 0.6	48.6** ± **0.3	47.5 ± 0.3	47.0 ± 0.8	46.5 ± 0.8	47.7 ± 1.2	52.1 ± 0.6*	46.8 ± 1.0	49.5 ± 0.8	50.9 ± 0.6
Progressive	48.9 ± 0.5	48.0 ± 0.4	49.4 ± 0.3	48.2 ± 0.4	48.1 ± 0.4	49.2 ± 0.5	49.3 ± 0.4	48.7 ± 0.3	48.1 ± 0.4	51.0 ± 0.4*	49.2 ± 0.4	49.9 ± 0.6
**pO**_**2**_** (mmHg)**												
Control	26.3 ± 0.2	26.0 ± 0.2	25.6 ± 0.2	26.1 ± 0.3	25.8 ± 1.1	24.5 ± 0.7	27.1 ± 0.3	24.9 ± 0.4	24.7 ± 1.0	26.3 ± 0.3	26.7 ± 1.0	25.6 ± 1.1
Acute-on-chronic	25.2 ± 0.5	24.5 ± 0.4	25.3 ± 0.7	26.7 ± 0.4	27.4 ± 0.7	25.5 ± 0.5	25.6 ± 0.5	23.0 ± 0.6	22.5 ± 0.6	25.8 ± 0.5	24.1 ± 0.4	21.6 ± 0.5
Progressive	25.6 ± 0.3	25.7 ± 0.4	23.8 ± 0.4	25.1 ± 0.3	25.5 ± 0.5	24.8 ± 0.4	25.4 ± 0.4	25.0 ± 0.4	24.8 ± 0.4	26.0 ± 0.4	25.5 ± 0.4	25.1 ± 0.4
**Hb (g/dL)**												
Control	8.6 ± 0.1	8.4 ± 0.1	8.3 ± 0.1	8.6 ± 0.1	7.9 ± 0.2	7.9 ± 0.2	9.0 ± 0.1	8.0 ± 0.1	8.1 ± 0.4	9.0 ± 0.1	8.2 ± 0.3	8.3 ± 0.3
Acute-on-chronic	8.2 ± 0.2	8.3 ± 0.2	7.8 ± 0.2	8.0 ± 0.2	7.7 ± 0.2	8.0 ± 0.2	8.0 ± 0.2	9.1 ± 0.2	8.9 ± 0.3	8.0 ± 0.2	9.4 ± 0.2	8.3 ± 0.2
Progressive	8.3 ± 0.1	8.9 ± 0.2	8.6 ± 0.1	8.1 ± 0.2	8.3 ± 0.2	8.1 ± 0.2	8.3 ± 0.2	8.2 ± 0.2	8.2 ± 0.2	8.4 ± 0.2	8.3 ± 0.2	8.5 ± 0.2
**ctO**_**2**_ **(mmol/L)**												
Control	3.6 ± 0.0	3.6 ± 0.0	3.5 ± 0.1	3.6 ± 0.1	3.2 ± 0.2	3.1 ± 0.1	3.8 ± 0.1	3.2 ± 0.0	3.1 ± 0.2	3.7 ± 0.1	3.5 ± 0.2	3.3 ± 0.2
Acute-on-chronic	3.3 ± 0.1	3.3 ± 0.1	3.2 ± 0.1	4.1 ± 0.2	4.2 ± 0.4	3.2 ± 0.1	3.2 ± 0.1*	3.2 ± 0.1	2.8 ± 0.1	3.3 ± 0.1	3.4 ± 0.1	2.5 ± 0.1
Progressive	3.5 ± 0.0	3.6 ± 0.1	3.2 ± 0.0	3.1 ± 0.0	3.3 ± 0.0	3.2 ± 0.1	3.2 ± 0.1*	3.2 ± 0.0	3.1 ± 0.1	3.3 ± 0.1	3.4 ± 0.1	3.3 ± 0.1
**BE (mmol/L)**												
Control	0.4 ± 0.2	0.7 ± 0.2	0.1 ± 0.2	0.3 ± 0.3	0.6 ± 0.8	0.6 ± 0.8	− 0.2 ± 0.2	− 0.7 ± 0.2	− 1.9 ± 1.5	− 0.9 ± 0.2	0.8 ± 0.5	0.6 ± 0.7
Acute-on-chronic	1.1 ± 0.4	1.2 ± 0.3	− 0.3 ± 0.6	0.6 ± 0.3	− 0.6 ± 0.2	− 0.5 ± 0.6	− 1.8 ± 0.3	− 1.2 ± 0.5	− 0.2 ± 0.4	− 1.4 ± 0.5	− 2.9 ± 0.4*	0.2 ± 0.2
Progressive	1.5 ± 0.3	1.2 ± 0.3	2.1 ± 0.2*	0.4 ± 0.3	0.7 ± 0.2	1.5 ± 0.3	0.9 ± 0.2	1.1. ± 0.3	0.0 ± 0.3	0.7 ± 0.2	1.3 ± 0.3	0.9 ± 0.3
**Lactate (mmol/L)**												
Control	0.8 ± 0.0	0.9 ± 0.0	0.9 ± 0.0	0.9 ± 0.0	0.9 ± 0.1	0.9 ± 0.1	0.8 ± 0.0	0.8 ± 0.0	0.9 ± 0.0	0.8 ± 0.0	0.8 ± 0.0	0.9 ± 0.1
Acute-on-chronic	0.8 ± 0.0	0.8 ± 0.0	1.2 ± 0.1	0.8 ± 0.0	0.8 ± 0.0	0.9 ± 0.0	0.8 ± 0.0	1.4 ± 0.1*	2.5 ± 0.2*	0.8 ± 0.0	1.2 ± 0.1	1.4 ± 0.1
Progressive	0.8 ± 0.0	0.9 ± 0.0	1.2 ± 0.1	0.8 ± 0.0	0.9 ± 0.0	0.9 ± 0.0	0.8 ± 0.0	0.8 ± 0.0	0.9 ± 0.0	0.8 ± 0.0	0.8 ± 0.0	0.9 ± 0.0
**Glucose (mmol/L)**												
Control	1.0 ± 0.0	1.1 ± 0.0	1.1 ± 0.0	1.0 ± 0.0	1.0 ± 0.1	1.1 ± 0.1	1.0 ± 0.0	0.9 ± 0.0	1.0 ± 0.1	1.0 ± 0.0	0.9 ± 0.1	1.0 ± 0.1
Acute-on-chronic	1.0 ± 0.0	1.0 ± 0.0	1.0 ± 0.0	1.0 ± 0.0	1.0 ± 0.0	1.0 ± 0.0	1.0 ± 0.0	1.0 ± 0.0	0.9 ± 0.0	1.0 ± 0.0	0.9 ± 0.0	0.9 ± 0.0
Progressive	0.9 ± 0.0	1.1 ± 0.0	1.1 ± 0.0	0.9 ± 0.0	1.1 ± 0.0	1.0 ± 0.0	0.9 ± 0.0	0.9 ± 0.0	1.0 ± 0.0	0.9 ± 0.0	0.9 ± 0.0	1.0 ± 0.0

*pCO2* partial pressure of carbon dioxide, *pO2* partial pressure of oxygen, *Hb* hemoglobin, *ctO*_*2*_ oxygen content, *BE* base excess.

*p < 0.05 vs. control.

**Table 2:** Arterial biochemistry from day 4 to day 10.

Day	Day 4			Day 5	Day 6	Day 7	Day 8	Day 9	Day 10
Time	Baseline	+ 2 h	+ 6 h	Baseline	Baseline	Baseline	Baseline	Baseline	Baseline
**pH**									
Control	7.35 ± 0.00	7.35 ± 0.01	7.34 ± 0.01	7.36 ± 0.00	7.35 ± 0.00	7.33 ± 0.00	7.33 ± 0.00	7.35 ± 0.00	7.33 ± 0.01
Acute-on-chronic	7.35 ± 0.00	7.34 ± 0.00	7.35 ± 0.00	7.36 ± 0.00	7.35 ± 0.00	7.33 ± 0.01	7.33 ± 0.01	7.34 ± 0.01	7.35 ± 0.00
Progressive	7.34 ± 0.00	7.35 ± 0.00	7.34 ± 0.00	7.34 ± 0.00	7.34 ± 0.00	7.34 ± 0.00	7.33 ± 0.00	7.33 ± 0.00	7.33 ± 0.00
**pCO**_**2**_** (mmHg)**									
Control	45.7 ± 0.4	45.3 ± 1.4	49.0 ± 0.7	46.5 ± 0.5	45.7 ± 0.4	48.7 ± 0.4	49.5 ± 0.2	48.8 ± 0.3	47.8 ± 1.2
Acute-on-chronic	46.0 ± 0.9	47.0 ± 1.3	47.8 ± 0.4	46.5 ± 0.3	46.0 ± 0.9	48.0 ± 0.6	48.5 ± 0.5	48.8 ± 0.4	48.5 ± 0.3
Progressive	48.8 ± 0.6	48.9 ± 0.6	49.1 ± 0.4	50.2 ± 0.6	48.8 ± 0.6	50.1 ± 0.5	47.9 ± 0.6	49.3 ± 0.6	50.3 ± 0.4
**pO**_**2**_** (mmHg)**									
Control	26.8 ± 0.2	25.9 ± 0.8	24.4 ± 1.5	27.7 ± 0.4	26.8 ± 0.2	24.9 ± 0.4	25.2 ± 0.4	25.9 ± 0.3	26.2 ± 1.1
Acute-on-chronic	26.7 ± 0.6	25.5 ± 0.5	25.2 ± 0.4	29.3 ± 0.7	26.7 ± 0.6	30.1 ± 0.9	28.6 ± 0.8	27.8 ± 0.9	27.0 ± 0.3
Progressive	24.4 ± 0.2	25.6 ± 0.5	24.7 ± 0.5	24.8 ± 0.3	24.4 ± 0.2	27.0 ± 0.4	25.8 ± 0.4	27.0 ± 0.3	25.8 ± 0.4
**Hb (g/dL)**									
Control	9.2 ± 0.2	8.0 ± 0.3	8.4 ± 0.3	9.5 ± 0.2	9.2 ± 0.2	9.8 ± 0.2	9.8 ± 0.2	9.9 ± 0.2	8.9 ± 0.5
Acute-on-chronic	8.1 ± 0.1	9.3 ± 0.2	8.4 ± 0.2	9.5 ± 0.4	8.1 ± 0.1	10.5 ± 0.5	10.1 ± 0.5	9.6 ± 0.5	10.6 ± 0.4
Progressive	8.4 ± 0.2	8.3 ± 0.2	8.3 ± 0.2	9.9 ± 0.6	8.4 ± 0.2	8.7 ± 0.2	9.0 ± 0.2	9.2 ± 0.2	9.2 ± 0.2
**ctO**_**2**_ **(mmol/L)**									
Control	3.7 ± 0.1	3.3 ± 0.2	3.2 ± 0.3	4.0 ± 0.1	3.7 ± 0.1	3.7 ± 0.1	3.8 ± 0.9	3.8 ± 0.1	3.6 ± 0.2
Acute-on-chronic	3.4 ± 0.1	3.6 ± 0.1	3.2 ± 0.1	3.9 ± 0.1	3.4 ± 0.1	4.2 ± 0.2	3.8 ± 0.1	4.0 ± 0.2	3.8 ± 0.1
Progressive	3.3 ± 0.1	3.4 ± 0.1	3.1 ± 0.1	3.4 ± 0.1	3.3 ± 0.1	3.7 ± 0.1	3.5 ± 0.1	3.8 ± 0.1	3.6 ± 0.1
**BE (mmol/L)**									
Control	− 1.2 ± 0.2	− 1.1 ± 0.8	0.1 ± 0.4	− 0.3 ± 0.3	− 1.2 ± 0.2	0.9 ± 0.2	0.7 ± 0.2	− 0.1 ± 0.2	− 0.9 ± 0.6
Acute-on-chronic	− 1.4 ± 0.4	− 1.2 ± 0.6	0.4 ± 0.1	0.3 ± 0.3	− 1.4 ± 0.4	− 0.6 ± 0.5	− 0.6 ± 0.6	0.6 ± 0.4	0.3 ± 0.2
Progressive	0.2 ± 0.3	1.0 ± 0.3	0.6 ± 0.1	1.1 ± 0.3	0.2 ± 0.3	1.0 ± 0.2	− 0.8 ± 0.2	− 0.2 ± 0.3	0.1 ± 0.2
**Lactate (mmol/L)**									
Control	0.7 ± 0.0	0.8 ± 0.1	0.9 ± 0.1	0.8 ± 0.0	0.7 ± 0.0	0.9 ± 0.0	0.9 ± 0.0	0.8 ± 0.0	0.8 ± 0.1
Acute-on-chronic	0.7 ± 0.0	0.8 ± 0.0	0.7 ± 0.0	0.7 ± 0.0	0.7 ± 0.0*	0.6 ± 0.0*	0.6 ± 0.0	0.7 ± 0.0	0.7 ± 0.0
Progressive	0.8 ± 0.0	0.9 ± 0.1	0.9 ± 0.0	0.8 ± 0.0	0.8 ± 0.0	0.8 ± 0.0	0.8 ± 0.0	0.8 ± 0.0	0.7 ± 0.0
**Glucose (mmol/L)**									
Control	0.8 ± 0.0	0.9 ± 0.1	1.0 ± 0.1	0.9 ± 0.0	0.8 ± 0.0	1.0 ± 0.0	1.0 ± 0.0	0.9 ± 0.0	0.8 ± 0.0
Acute-on-chronic	1.0 ± 0.0	0.9 ± 0.0	1.0 ± 0.0	0.9 ± 0.0	1.0 ± 0.0	0.7 ± 0.0*	0.7 ± 0.0*	0.7 ± 0.0	0.7 ± 0.0
Progressive	0.9 ± 0.0	0.9 ± 0.0	0.9 ± 0.0	0.9 ± 0.0	0.9 ± 0.0	0.9 ± 0.0	0.8 ± 0.0*	0.8 ± 0.0	0.8 ± 0.0

*pCO2* partial pressure of carbon dioxide, *pO2* partial pressure of oxygen, *Hb* hemoglobin, *ctO*_*2*_ oxygen content, *BE* base excess.

*p < 0.05 vs. control.

### Progressive LPS infusions

The progressive LPS protocol was associated with only transient changes after the start of infusions. FHR increased in the progressive LPS group compared to controls from 6 to 9 h (p = 0.011, Fig. 2) after the start of progressive LPS infusions at the lowest rate (0.2 µg/kg/24 h). Thereafter, there were no changes in FHR compared to controls. There were no changes in MAP or nuchal EMG throughout the study period (Fig. 2).

**Figure 3:**
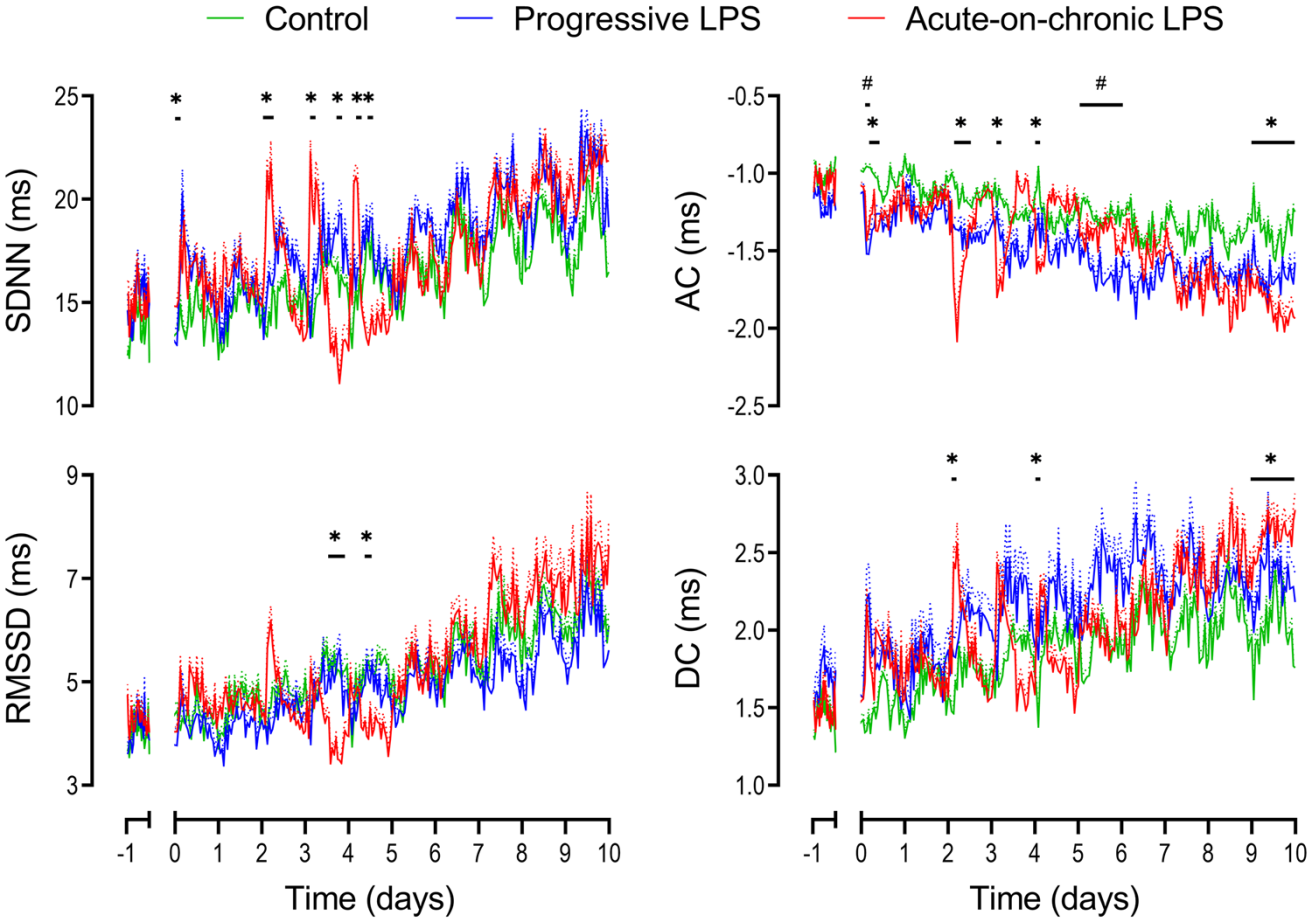
Changes in time domain measures, acceleration capacity (AC) and deceleration capacity (DC) during and after LPS infusions. Time domain measures included the standard deviation of RR intervals (SDNN) and the root mean square of successive RR intervals (RMSSD). All LPS infusions were started on day 0 and continued for a total of 5 days, fetuses were studied for a further 5 days after the end of infusions. Groups shown are control (green, n = 10), progressive LPS (blue, n = 8) and acute-on-chronic LPS (red, n = 9). Data are shown as 1 h mean ± SEM (mean is displayed as solid lines, SEM is displayed as dotted lines). *P < 0.05 control vs. acute-on-chronic LPS, ^#^P < 0.05 control versus progressive LPS.

Only subtle changes in measures of FHRV were observed, all shortly after the start onset of LPS infusions. There was a decrease in both acceleration capacity from 3 to 6 h (p = 0.034 vs controls, Fig. 2) and Sample Entropy from 6 to 12 h (p = 0.033, Fig. 3).

After the end of the LPS infusions at 120 h, the progressive LPS group showed a further reduction in acceleration capacity compared to controls from 120 to 144 h (p = 0.049, Fig. 3). No further changes in either physiological parameters or measures of FHRV were observed in the 5 d after the end of progressive LPS infusions (Fig. 2).

### Acute on chronic LPS infusions

The initial two days of low-dose LPS infusions in the acute-on-chronic group (0.1 and 0.2 µg/kg/24 h) were not associated with any change in FHR, MAP or nuchal EMG activity (Fig. 2). Subtle changes in measures of FHRV were observed early after the initial onset of LPS infusions (0.1 µg/kg/24 h). Both VLF (p = 0.01) and LF (p = 0.047) were increased from 0–3 h after onset of LPS infusions compared to controls (Fig. 5). SDNN increased from 3 to 6 h (p = 0.029) and acceleration capacity was decreased from 3 to 9 h (p = 0.012) after the onset of LPS infusion in the acute-on-chronic group compared to controls (Fig. 3). No further changes in FHRV were observed when LPS infusions were increased at 24 h (0.2 µg/kg/24 h).

The three 1 µg LPS boluses were associated with hypotension, tachycardia and reduced nuchal EMG activity which became progressively attenuated with each bolus (Fig. 2). All time points in this section are described relative to the timing of each bolus. MAP fell in the acute-on-chronic group compared to controls after the first (3–12 h, p = 0.001) and second LPS boluses (6–12 h, p = 0.006). After the third LPS bolus, an initial period of increased MAP was observed (0–3 h, p = 0.033) but no significant hypotension was observed. FHR increased in the acute-on-chronic group compared to controls after the first (0–15 h, p = 0.01), second (3–12 h, p = 0.002) and third LPS boluses (3–9 h, p = 0.013). After resolution of the initial tachycardia after the second LPS bolus, FHR fell and was reduced in the acute-on-chronic group compared to controls from 18 to 24 h (p = 0.027, Fig. 2).

Nuchal EMG activity was decreased after the first LPS bolus from 0 to 9 h in the acute-on-chronic group compared to controls (p = 0.01). After the second LPS bolus, nuchal EMG activity was initially reduced from 0 to 3 h (p = 0.038) and subsequently increased from 21 to 24 h (p = 0.019) in the acute-on-chronic group compared to controls (Fig. 2).

### Fetal heart rate variability after LPS boluses

LPS boluses were associated with a marked biphasic pattern across multiple measures of FHRV, generally characterized by an initial increase followed by suppression of FHRV. With subsequent boluses, the initial increase appeared to become attenuated while the secondary phase of FHRV suppression was only observed after the second and third bolus. All time points in this section are described relative to the timing of each bolus.

**Figure 4:**
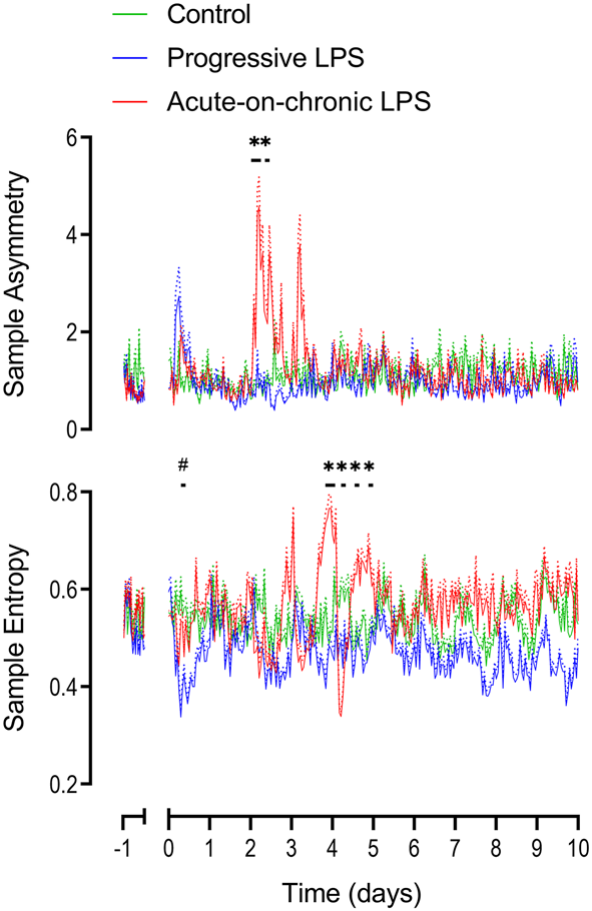
Changes in sample entropy and sample asymmetry during and after LPS infusions. All LPS infusions were started on day 0 and continued for a total of 5 days, fetuses were studied for a further 5 days after the end of infusions. Please note that sample entropy and sample asymmetry are not related indices and are grouped together for convenience. Groups shown are control (green, n = 10), progressive LPS (blue, n = 8) and acute-on-chronic LPS (red, n = 9). Data are shown as 1 h mean ± SEM (mean is displayed as solid lines, SEM is displayed as dotted lines). *P < 0.05 control vs. acute-on-chronic LPS, ^#^P < 0.05 control versus progressive LPS.

**Figure 5:**
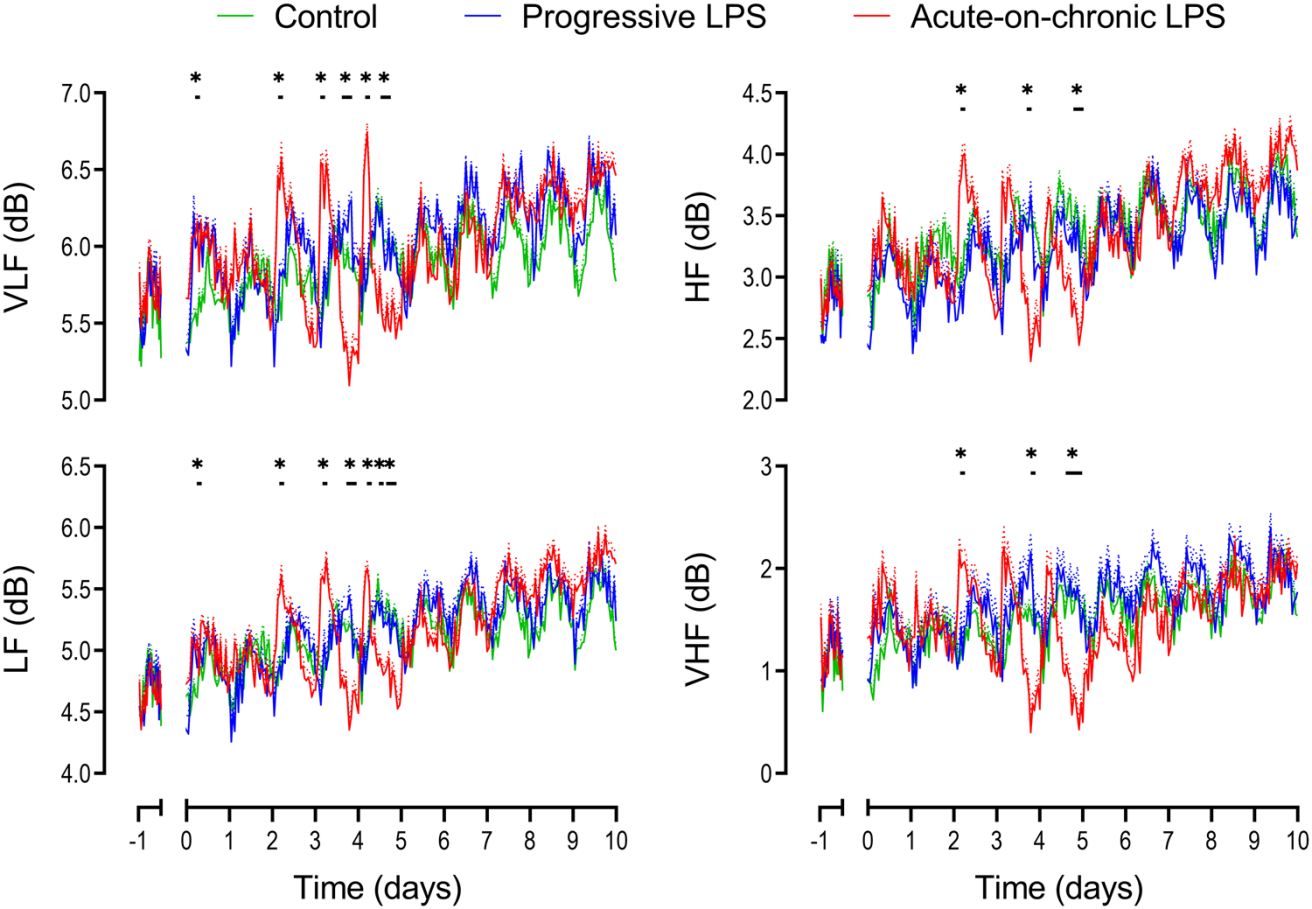
Changes in frequency domain measures during and after LPS infusions. Frequencies presented include very low frequency (VLF, 0.0033–0.04 Hz), low frequency (LF, 0.04–0.15 Hz), high frequency (HF, 0.15–0.4 Hz) and very high frequency activity (VHF, 0.4–1.5 Hz). All measures were natural log transformed for presentation. All LPS infusions were started on day 0 and continued for a total of 5 days, fetuses were studied for a further 5 days after the end of infusions. Groups shown are control (green, n = 10), progressive LPS (blue, n = 8) and acute-on-chronic LPS (red, n = 9). Data are shown as 1 h mean ± SEM (mean is displayed as solid lines, SEM is displayed as dotted lines). *P < 0.05 control vs. acute-on-chronic LPS.

After the first LPS bolus the increase in FHRV was characterized by an increase in SDNN (0–6 h, p = 0.016), VLF (3–6 h, p = 0.003), LF (3–6 h, p = 0.016), HF (3–6 h, p = 0.017), VHF (3–6 h, p = 0.049), deceleration capacity (3–6 h, p = 0.043) and sample asymmetry (0–6 h, p = 0.027 and 9–12 h, p = 0.045) and a decrease in acceleration capacity (0–9 h, p = 0.006) compared to controls (Figs. 3, 4, 5). After the second LPS bolus, increased FHRV was seen with an increase in SDNN (3–6 h, p = 0.022), VLF (3–6 h, p = 0.006), LF (3–6 h, p = 0.002), and a decrease in acceleration capacity (0–3 h, p = 0.031, compared to controls, Figs. 3 and 5). After the third LPS bolus, increased FHRV was characterized by an increase in SDNN (3–6 h, p = 0.042), VLF (3–6 h, p = 0.002), LF (3–6 h, p = 0.018), deceleration capacity (0–3 h, p = 0.036) and a decrease in acceleration capacity (0–3 h, p = 0.002) and sample entropy (3–6 h, p = 0.002) compared to controls (Figs. 3, 4, 5).

FHRV suppression was not seen after the first LPS bolus. However, after the second LPS bolus there was secondary suppression of FHRV characterized by a decrease in SDNN (15–18 h, p = 0.03), RMSSD (12–21 h, p = 0.031), VLF (15–21 h, p = 0.031), LF (15–21 h, p = 0.021), HF (18–21 h, p = 0.019), VHF (18–21 h, p = 0.32) and an increase in Sample Entropy (18–24 h, p = 0.025) compared to controls (Figs. 3, 4, 5). After the third bolus, a decrease in SDNN (9–12 h, p = 0.004), VLF (9–15 h, p = 0.031), LF (9–12 h, p = 0.023 and 18–24 h, p = 0.04), HF (18–24 h, p = 0.035), VHF (15–24 h, p = 0.021) and an increase in Sample Entropy (18–21 h, p = 0.027) was observed compared to controls (Figs. 3, 4, 5).

During the 5 d period of recovery, from day 6 to 10 inclusive, after the end of the LPS infusions (i.e. 24 h after the third LPS bolus), the majority of measures of FHRV in the acute-on-chronic group remained no different to controls. On the final day of recovery (216–240 h after the initial onset of low-dose infusions), a decrease in acceleration capacity (p = 0.037) and an increase in deceleration capacity (p = 0.033) was observed in the acute-on-chronic group compared to controls (Fig. 3).

## Discussion

The present study demonstrates that there are markedly reduced fetal physiological responses to gradually worsening fetal inflammation (the progressive LPS protocol) compared to episodes of rapid, severe exposure to inflammation superimposed on a low background of inflammation (the acute-on-chronic LPS protocol). The magnitude of changes in multiple measures of FHRV were in turn highly related to the magnitude of the physiological responses associated with each protocol, even though the progressive protocol delivered more LPS (6.2 µg over 5 days) than the acute on chronic protocol (4.125 µg over 5 days). Thus, the different FHRV patterns reflect the pattern of LPS exposure rather than the amount given. We have previously shown that both these protocols are associated with neural injury^23,24,29^, suggesting that changes in FHRV may be a useful biomarker of acute cardiovascular dysfunction, but not necessarily of inflammation-induced neural injury.

The present findings should be interpreted in conjunction with the patterns of inflammatory cytokine level observed in each protocol. Concentrations of interleukin (IL)-6, IL-8, IL-10 and tumor necrosis factor in these cohorts have previously been quantified using assays validated in fetal sheep, please see the original manuscripts for full descriptions^21,23,24,28,29^. Unfortunately, the cytokine analyses in the two cohorts were performed using different methodologies and so cannot be directly compared, however, broad comparisons are still appropriate. Focusing on changes in the key pro-inflammatory marker IL-6; in the acute-on-chronic protocol a marked increase in IL-6 was observed at 2 and 6 h after the first LPS bolus (1 µg) on day 3, which resolved by 24 h after the bolus. The subsequent two boluses on days 4 and 5 showed a markedly attenuated IL-6 response^21,23,28,29^. In the progressive LPS group, a mild but transient increase in IL-6 was observed 6 h after the initial onset of LPS infusions on day 1. A second more sustained rise in IL-6 was observed on the morning of day 4 (when the LPS infusion rate was increased from 0.8 to 1.6 µg/kg/24 h) until the morning of day 6 when the LPS infusion (at 3.2 µg/kg/24 h) finished^24^. The magnitude of the increase in IL-6 after LPS boluses in the acute-on-chronic group was approximately 5 times higher than the peak observed in the progressive LPS group.

A key aim of the present study was to investigate whether in-depth FHRV analysis could identify potential biomarkers for mild/subclinical fetal infections. The present study demonstrated transient changes at the start of the lowest dose infusion in the progressive LPS group. On the first day of low-dose infusions, there was tachycardia from 6 to 9 h in association with a decrease in both acceleration capacity and sample entropy. This combination suggests a relative reduction in accelerations and a less complex FHR trace. Thereafter, we observed no further changes across multiple measures of FHRV despite this group receiving a higher overall dose of LPS than the acute-on-chronic LPS group. This is particularly surprising considering that the progressive LPS group received 3.2 µg over the final day of infusion, in comparison to the highest daily dose of 1.25 µg administered to the acute-on-chronic group. Interestingly, after the end of the progressive LPS infusions there was a modest reduction in acceleration capacity for the first 24 h, suggesting a small relative reduction in the number or amplitude of accelerations. By contrast, we observed marked, biphasic changes across multiple measures of FHRV after exposure to high-dose (1 µg) LPS boluses in the acute-on-chronic group. Consistent with previous findings, the biphasic changes appear to be temporally associated with the onset and recovery of arterial hypotension^21,22^, as discussed below.

Although we observed no changes in physiological parameters or measures of FHRV for the majority of the progressive LPS protocol, we have shown that there was a sustained increase in serum IL-6, in particular over the final two days of LPS infusions^24^. At the end of the experiment, the brains of these fetal sheep showed evidence of white matter injury and diffuse astrogliosis based on both histological and MRI-based findings^24^. Therefore, impaired neurodevelopment may still occur following progressive intrauterine inflammation without overt signs of inflammation-induced cardiovascular deterioration. This emphasizes the need for other biomarkers for milder/subclinical intrauterine infection/inflammation.

### FHRV in the acute-on-chronic group

In the acute-on-chronic group, the low-dose infusions were associated with a similar time course and pattern of changes in multiple FHRV parameters compared to the progressive LPS group. Interestingly though, more of these changes reached statistical significance which we speculate reflects slight differences in the susceptibility of individual fetuses to LPS. Indeed, we have previously found that there can be marked variability to the response to LPS^21,22^. In the acute-on-chronic group, we observed an increase in SDNN, VLF and LF and a decrease in acceleration capacity variably between 0 and 9 h after the start of low-dose infusions, suggesting a broad but predominantly low frequency increase in FHRV, but reduced FHR accelerations.

LPS boluses in the acute-on-chronic group were associated with hypotension and tachycardia. As previously reported, hypotension after high-dose LPS boluses is likely driven by peripheral vasodilation and impaired myocardial contractility in association with increased IL-6 and tumor necrosis factor levels^21,24,29^. These responses became attenuated with repeated boluses, consistent with endotoxin tolerance^45^. Although tolerance is not fully understood, in neonatal rodents it is associated, in part, with up-regulation of corticosterone^46^.

Biphasic changes in multiple measures of FHRV occurred after the LPS boluses, similar to our previous studies that examined time domain measures^21,22^. The acute increase in measures of FHRV after bolus LPS administration is in turn consistent with the original findings of Blad and colleagues in both preterm and term fetal sheep^47^. Durosier, Herry and colleagues reported complex changes in multiple parameters of FHRV after smaller LPS boluses (400 ng) in term fetal sheep, and suggested that composite measures of highly selected, predominantly non-linear indices may correlate with IL-6 levels^48,49^. Unfortunately, the characteristics of individual parameters were not clearly presented in these studies, precluding comparison to the present work.

After LPS boluses in the present study, there was an initial increase in most FHRV measures, suggesting an overall increase in FHRV. This excitatory phase preceded and overlapped with the development of arterial hypotension and tachycardia, and was associated with decreased nuchal EMG activity, suggesting it was not related to fetal body movements. This phase coincided with the timing of peak serum cytokine levels that we have previously reported in similar cohorts^21,23^. LPS is not able to cross the blood–brain barrier^50^, and therefore it is likely that the increased FHRV observed at this time represents autonomic dysfunction secondary to increased systemic pro-inflammatory cytokines^51^. Further, the cholinergic anti-inflammatory response may have been stimulated, promoting increased parasympathetic outflow^52,53^.

During the first six hours after the first bolus of LPS there was an increase in SDNN (a measure of total FHRV), and an increase in all four frequency domain measures (VLF, LF, HF and VHF) suggesting a broad, non-specific increase in FHRV. Interestingly, there was an increase in deceleration capacity and a decrease in acceleration capacity, with increased sample asymmetry. All three of these findings suggest an inflammation-induced reduction in FHR accelerations and a corresponding increase in FHR decelerations^43^. This is consistent with our previous finding of increased sample asymmetry after LPS boluses, in association with the visual appearance of shallow, transient FHR decelerations^22^. Similar patterns have been reported in developing neonatal sepsis^13,17,43^. These patterns progressively became attenuated after the second and third LPS boluses, particularly, the transient increases in HF and VHF.

Following the resolution of both the initial excitation phase and subsequent, slower resolution of arterial hypotension, a second phase of relative suppression of multiple measures of FHRV was observed. This phase was not observed after the first LPS bolus, suggesting that it may have been masked by the more pronounced and more prolonged initial excitatory phase at that time. There was a decrease in SDNN, RMSSD (second bolus only), VLF, LF, HF and VHF, supporting a broad reduction across all frequencies of FHRV. We also observed an increase in Sample Entropy, suggesting increased complexity of the FHR trace. This finding was unexpected, considering that neonatal sepsis has been associated with decreased Sample Entropy^54^. By contrast, in the present study there was only brief suppression of Sample Entropy during the initial excitation phase after the third bolus.

This period of broad suppression of FHRV was observed following resolution of arterial hypotension, at a time when systemic inflammatory markers are likely declining^21,23^. Suppression of FHRV strongly suggests relative autonomic quiescence or withdrawal. Although suppression of FHRV is most often considered to be an ominous sign, in the present study suppression of FHRV did not occur until after resolution of cardiovascular dysfunction, at time when levels of pro-inflammatory cytokines are falling. There is some evidence that LPS-induced inflammation can directly impair the function of the sinoatrial node^55^. However, the observation that there were largely no changes in FHRV in the progressive-LPS group despite a higher overall dose of LPS suggests that this mechanism did not materially contribute to suppressed FHRV.

The present study represents the longest analysis of FHRV during recovery after fetal inflammation, to the best of our knowledge. Previous studies by our team and others only assessed FHRV for 4 days, or less after induction of fetal inflammation^21,22,47,49^. Throughout most of the five day recovery period after the end of LPS infusions, almost all FHRV parameters in both the acute-on-chronic and progressive LPS groups returned to control levels, demonstrating that the periods of suppressed FHRV after LPS were unlikely to reflect permanent damage to autonomic centers, even though both the present LPS protocols and many others are associated with neuroinflammation and impaired white and grey matter development^23,24,56,57^. This study suggests that inflammation-induced brain injury per se is not associated with marked alterations to FHRV.

This is broadly consistent with evidence that even severe forebrain injury does not affect FHRV, supporting the hypothesis that the hindbrain is essentially the sole origin of HRV in the fetus^58^. Of particular interest, one study in preterm fetal sheep has shown that the brainstem was relatively spared from LPS-induced injury and inflammatory changes despite LPS triggering cerebellar injury^56^. Nonetheless, it is reasonable to note that the acute-on-chronic group showed a late, modest decrease in acceleration capacity and a modest increase in deceleration capacity over the final 24 h of recovery, suggesting that inflammation may be associated with subtle changes to the maturation of FHR trace. This was not observed in the progressive LPS group despite the higher total LPS dose, highlighting the importance of the magnitude of the associated fetal inflammatory response.

Clinical translation of our findings is limited at present by the low-resolution of Doppler-based FHR monitoring, which does not allow for the assessment of true beat-to-beat FHRV^59^. Basic time-domain measures including SDNN in this report, or the mean-minute range as we have previously reported after acute-on-chronic LPS^21^, provide the best approximation of the visual assessment of FHR patterns. It is additionally likely to be feasible to visually identify an ‘asymmetric’ FHR trace (characterized by a predominance of brief decelerations and a relative absence of accelerations) as observed after high-dose boluses in the acute-on-chronic LPS group via current visual analysis. Nonetheless, newer technology allowing non-invasive measurement of the true fetal ECG are increasingly becoming available which will ultimately allow more in-depth analysis of FHRV in real-time^59^ and further translation of these findings.

### Conclusions and perspectives

The present study sought to identify potential FHR biomarkers that may help identify subclinical intrauterine infections. Unfortunately, our findings suggest that the FHR trace remains largely unaltered unless the fetus mounts a substantial inflammatory response. The corollary of this is that chronic, slowly worsening levels of fetal inflammation seem to be unlikely to be detectable by even in-depth FHRV analysis, despite being associated with neuroinflammation and impaired neurodevelopment^24^. The limited effect on FHRV observed in the progressive LPS group may in part help explain the recent finding that a sizeable portion of neonates with culture-proven late-onset sepsis were not identified by HRV analysis via the heart rate characteristic system^18^.

The present study provides additional information about the dynamic, biphasic alterations to FHRV during an acute fetal inflammatory response, which may help identify biomarkers for severe intrauterine infections. In particular, the findings suggest that FHRV measures that give clues as to the relative proportions of accelerations and decelerations within the FHR trace may be particularly useful, particularly sample asymmetry, acceleration capacity and deceleration capacity, which were acutely altered before and during the onset of arterial hypotension. Consistent with data from neonatal sepsis, we propose that asymmetric FHR traces due to a relative reduction in accelerations and a relative increase in decelerations represents the most promising biomarker of the cardiovascular and autonomic dysfunction associated with severe, rapidly worsening intrauterine infection and may warn of evolving arterial hypotension.
